# True 3q Chromosomal Amplification in Squamous Cell Lung Carcinoma by FISH and aCGH Molecular Analysis: Impact on Targeted Drugs

**DOI:** 10.1371/journal.pone.0049689

**Published:** 2012-12-06

**Authors:** Matteo Brunelli, Emilio Bria, Alessia Nottegar, Sara Cingarlini, Francesca Simionato, Anna Caliò, Albino Eccher, Claudia Parolini, Antonio Iannucci, Eliana Gilioli, Serena Pedron, Francesco Massari, Giampaolo Tortora, Ioana Borze, Sakari Knuutila, Stefano Gobbo, Antonio Santo, Luca Tondulli, Francesco Calabrò, Guido Martignoni, Marco Chilosi

**Affiliations:** 1 ISH Molecular Lab, Department of Pathology and Diagnostic, Azienda Ospedaliera Universitaria Integrata di Verona, University of Verona, Verona, Italy; 2 Medical Oncology, University of Verona, Verona, Italy; 3 Pathology Unit, Azienda Ospedaliera Universitaria Integrata di Verona, Ospedale Civile Maggiore, Verona, Italy; 4 Haartman Insitute, University of Helsinki, Helsinki, Finland; 5 Oncology Unit, Azienda Ospedaliera Universitaria Integrata di Verona, Ospedale Civile Maggiore, Verona, Italy; 6 Thoracic Surgery Unit, Azienda Ospedaliera Universitaria Integrata di Verona, Ospedale Civile Maggiore, Verona, Italy; University of Porto, Portugal

## Abstract

Squamous lung carcinoma lacks specific “ad hoc” therapies. Amplification of chromosome 3q is the most common genomic aberration and this region harbours genes having role as novel targets for therapeutics. There is no standard definition on how to score and report 3q amplification. False versus true 3q chromosomal amplification in squamous cell lung carcinoma may have tremendous impact on trials involving drugs which target DNA zones mapping on 3q. Forty squamous lung carcinomas were analyzed by FISH to assess chromosome 3q amplification. aCGH was performed as gold-standard to avoid false positive amplifications. Three clustered patterns of fluorescent signals were observed. Eight cases out of 40 (20%) showed ≥8 3q signals. Twenty out of 40 (50%) showed from 3 to 7 signals. The remaining showed two fluorescent signals (30%). When corrected by whole chromosome 3 signals, only cases with ≥8 signals maintained a LSI 3q/CEP3 ratio >2. Only the cases showing 3q amplification by aCGH (+3q25.3−3q27.3) showed ≥8 fluorescent signals at FISH evidencing a 3q/3 ratio >2. The remaining cases showed flat genomic portrait at aCGH on chromosome 3. We concluded that: 1) absolute copy number of 3q chromosomal region may harbour false positive interpretation of 3q amplification in squamous cell carcinoma; 2) a case results truly “amplified for chromosome 3q” when showing ≥8 fluorescent 3q signals; 3) trials involving drugs targeting loci on chromosome 3q in squamous lung carcinoma therapy have to consider *false* versus *true* 3q chromosomal amplification.

## Introduction

Squamous cell cancer accounts for about thirty per cent of lung cancer [1]. Unlike adenocarcinomas of the lung among which the increasing genomic characterization has simultaneously led to increase their biological portrait [1], [2], [3], squamous cell carcinoma remains a disease now bereft of a molecular targeted profile. This leads to consider the need for studies that enhance the molecular changes that, like EGFR, K-ras and ALK genes for lung adenocarcinoma, at prognostic and/or predictive levels may give chances for an effective therapeutic response in these patients [4]. In squamous cell carcinoma amplification of chromosome 3q region has been observed as the most common genomic aberration [5]. 3q amplification is important in the tumorigenesis of squamous cell carcinoma but not necessarily in adenocarcinoma, thus the 3q amplification itself also represents one of the most striking sensitive differences between squamous cell carcinoma and adenocarcinoma of the lung [6], [7]. Moreover, in addition to the diagnostic setting, the 3q region harbors many potential targeted genes, some of which already showed promising value as biomarkers in selecting patients for specific drugs, such as anti-PI3K and -SOX2 targeted therapies [8]. Actually, squamous lung carcinoma remains a neoplastic tissue orphan of specific “ad hoc” therapies, thus there is a need to identify new molecular and cytogenetic biomarkers usefull in selecting patients for new drugs and in designing new clinical trials [9], [10].

The interphase in situ hybridization technique is becoming a routinely available standard molecular assessment requested to reference Pathology Labs, due i.e. to the value of the Her-2 gene in breast, gastric cancers, 1p/19q in oligodendrogliomas or EGFR and ALK genes in lung adenocarcinoma [11], [12], [13], [14], [15]. Differently for the aforementioned biomarker assessment on which standard guidelines have been proposed, there is no clear definition on how to analyze analytically 3q chromosomal abnormalities and how to initially determine and finally score and report 3q amplification. The lack of a precise method for calculation may lead to a different interpretation of signals among different labs and across different studies; reading and interpreting the ISH assay reinforce the need for standardised testing procedures. In squamous cell carcinomas, at both cytogenetic and molecular levels, reports often do not distinguish chromosomal amplification due to an increase of the locus specific region 3q (and the degree) or to the entire chromosome 3. Polyploidy and genetic instability may bring false positive interpretation of 3q amplification.

In the actual study we sought to evaluate the subtypes of genotypic abnormalities of the entire chromosome 3 and the distal locus specific 3q that maps the SOX-2 and PI3CA genes in a serie of squamous lung carcinoma [16], [17], [18], [19], [20], [21], [22], [23], by weighting different chromosomal anomalies mapped by fluorescent ISH (FISH) and aCGH front techniques on routinely available formalin-fixed neoplastic tissue.

Finally, we inizialize a scoring for the assessment of 3q amplification, in order to provide a tool to support the clinical laboratories either for the diagnostic goal either for selection to clinical trials encountering inhibitors targeting the 3q region such as anti- PI3CA or –SOX2 targeted drugs.

## Materials and Methods

### Ethic Statements

We used tissue samples from human participants. All tissue blocks have been previously declaired to be available for the purposes of the actual study by the Istitutional Review Board (study conducted according to the principles expressed in the Declaration of Helsinki). Our institutional review board and the ethics committee approved the original human work that produced the tissue samples (Prof. Marco Chilosi, Director of the Pathology Unit, Azienda Ospedaliera Integrata di Verona, Verona, Italy and Prof. Aldo Scarpa, Director of the Department of Pathology and Diagnostic). All processing in obtaining the material has been performed after a written informed consent.

Full name Ethic/Institutional Review Board: Prof. Marco Chilosi, Prof. Aldo Scarpa, Prof. Francesco Calabrò, Prof. Guido Martignoni, Nucleo Ricerca&Innovazione.

### Tumour Selection

Fourty squamous lung carcinomas were recruited from the LUNG VERONA DATABASE files, Italy. All samples per tumour were reviewed by a pathologist expert in the field (MC) and an appropriate sample containing at least 90% neoplastic cells has been selected for the interphase cytogenetic and molecular studies.

### Immunophenotypical analysis

Immunophenotyping was performed by using Δn-p63 (rabbit, Oncogene), CK5 (XM26, Novocastra), CK7 (OV-TL12/30, Biogenex), TTF-1 (8G7G3/1, Dako), Napsin A (TMU-A d02, Arp), and SOX-2 (rabbit, Seven Hills).

### Fluorescence in situ hybridization analysis (FISH) analysis

Interphase cytogenetic fluorescence in situ hybridization analysis was performed using commercially available telomeric specific probe (TelVysion 3q, D3S4560) mapping on chromosome 3q telomere (SpectrumOrange LSI, Vysis-Abbott) and a centromeric (alpha-satellite DNA, SpectrumGreen) probe mapping on 3p11-q11. FISH procedure was developed according to previous reports from our group [24].

5 µm sections were cut from paraffin-embedded blocks. The procedure of FISH has been performed as detailed in previous manuscript [24].

Centromeric probes for chromosomes 3 and locus specific probes for 3q (Vysis-Abbott, Olympus, Rome, Italy) were used. Each probe was diluted 1∶10 in tDenHyb2 buffer (Insitus, Albuquerque, NM). Ten microliters of diluted probe were applied to each slide and cover slips were placed over the slides. Denaturation was achieved by incubating the slides at 80°C for 10 minutes in a humidified box; then hybridization was done at 37°C for 16 hours. The cover slips were then removed and the slides were immersed at room temperature in 0.5 XSSC for 2 minutes and in 2 XSSC for 2 minutes. The slides were air dried and counterstained with 10 µl DAPI/Antifade (DAPI in Fluorguard, 0.5 µg/ml, Insitus, Albuquerque, NM).

The slides were examined using an Olympus BX61 (Germany) with appropiate filters for SpectrumOrange (Tel 3q, Vysis-Abbott), SpectrumGreen (centromeric probe 3, Vysis-Abbott), and the UV Filter for the DAPI nuclear counterstain. The signals were recorded with a CCD camera (CytoVysion, Olympus Berlin, Germany and Fluo/D-SIGHT Menarini/Visia Imaging, Italy).

Fluorescent in situ signals were evaluated on carcinomatous and normal pulmonary adjacent parenchyma.

Fluorescent 3q signals was initially scored per each case. These cases were corrected per the score of chromosome 3 signals (LSI 3q/CEP3 >2 “amplified”). From 60 to 250 neoplastic nuclei were assessed per tumours, similarly per adjacent normal parenchyma.

### Array CGH and data analysis

Genomic DNA was isolated using QIAamp DNA mini kit (Qiagen Nordic, Finland) and quantified on the NanoDrop Spectrophotometer (NanoDrop Technologies Inc., DE, USA). The quantities and qualities of the DNA allowed us to continue with the analyses. As reference was used DNA from pooled peripheral blood leucocytes of healthy males. With Agilent Human 44K array format containing around 44 000 oligonucleotide probes, covering coding and noncoding genome region (Agilent Technologies, Santa Clara, CA), we screened for copy number alteration 6 tumours (2 tumours per each clustered categories detected at FISH analysis). Briefly, 1.5 µg of tumor and reference DNA were digested, labeled and hybridized according with the Agilent Protocols (version 4). The digested DNAs were labeled by random priming with Cy3-dUTP (reference DNA) and Cy5-dUTP (patient DNA) by use of the Agilent Labelling Kit, after which the labelled DNAs were purified. The resolution of the fragments analyzed varied from several Kb to <1 Mb depending upon the case selected.

The array images obtained after scanning (high resolution Agilent scanner) were processed with the Feature Extraction software (version 10.5), and the output data files were analyzed with the Agilent Genomic Workbench. To identify copy number alterations we used Aberration Detection Method 2 (ADM-2) algorithm and to exclude the small variances in the data we set up a custom aberration filter identifying an alterations in copy number if minimum of 8 probes gained or lost to be present and with minimum absolute average log ratio for region to be 0.5. The region with small copy number variations were excluded by comparing and visualizing the copy number variant regions tool of the Genomic workbench software.

## Results

### Tumours

**Figure 1:**
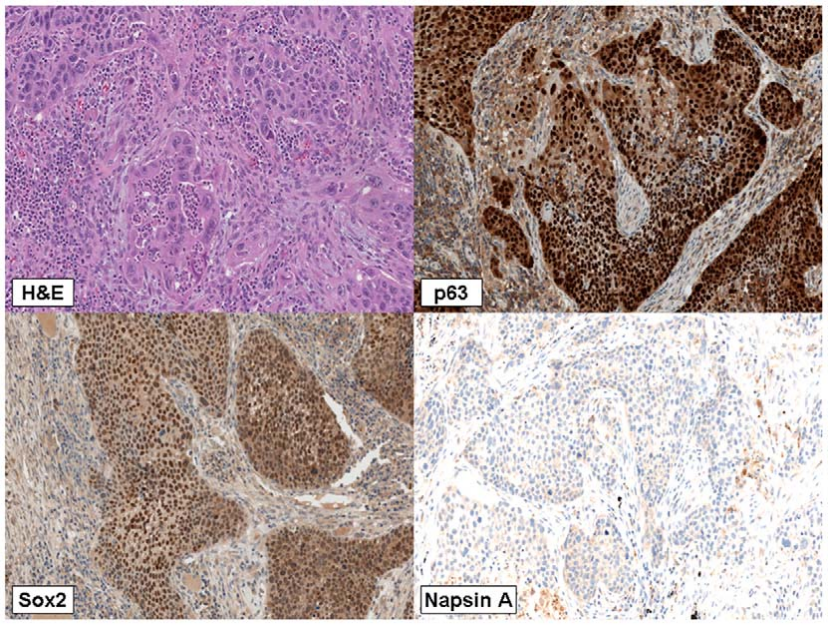
Squamous cell lung carcinoma. Squamous differentiation of cancerous neoplastic cells (a) showing immunoexpression of p63 (b), SOX2 at nuclear level (c), and absence of Napsin-A (d).

All carcinomas showed pure squamous morphology (Fig. 1a); 11 cases (27,5%) displayed a well, 25 (62,5%) moderate, 4 (10%) (scarse) degree of differentiation. Thirty carcinomas staged Ia, eight Ib, one IIa and one IIb.

### Immunophenotypical findings

Immunophenotipically p63 and SOX2 were expressed in all cases (100%) (respectively Fig. 1b, 1c); 4 cases (11%) expressed CK7 whereas TTF-1 was always absent. All the cases were also negative for Napsin A (Fig. 1d).

### FISH findings

**Figure 2:**
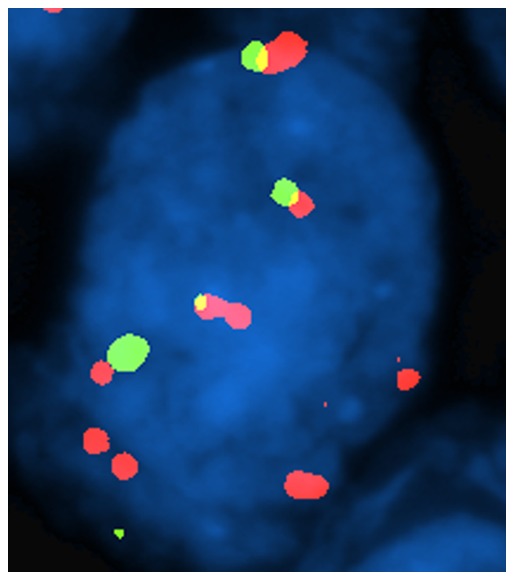
FISH findings in squamous cell neoplastic nuclei. 3q amplification.

**Figure 3:**
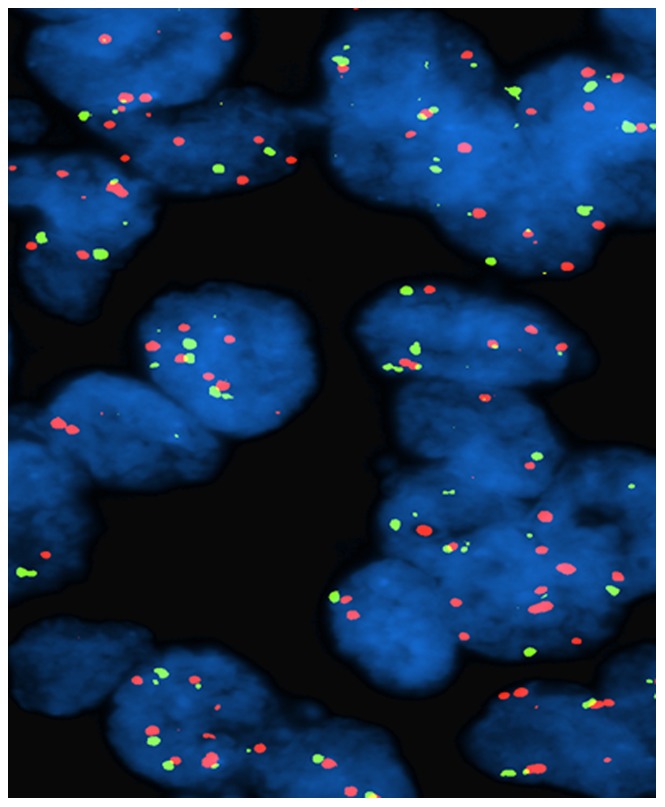
FISH findings in squamous cell neoplastic nuclei. Nuclei with polysomy of chromosome 3 without 3q amplification (polyploidy).

**Figure 4:**
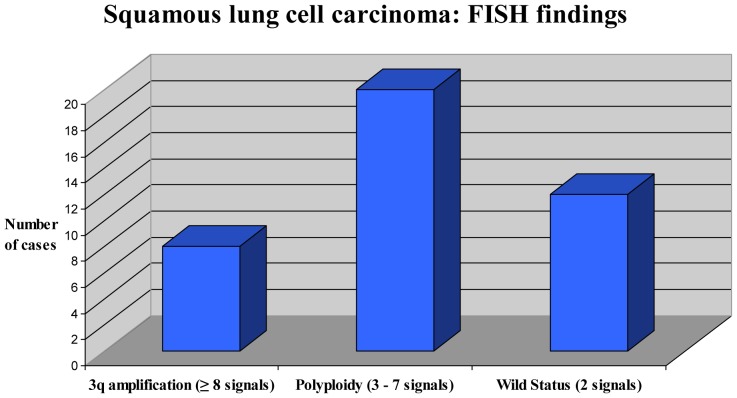
Squamous lung cell carcinoma. FISH scoring clustered into three groups after matching aCGH validation analysis. Notably, cases showing from 3 to 7 fluorescent signals displayed no 3q amplification after aCGH analysis, thus showing false 3q amplification by FISH. Cases showing ≥8 fluorescent signals displayed at aCGH analysis true 3q amplification.

We observed three clustered patterns of fluorescent signals (Fig. 4). Eight cases out of 40 (20%) showed ≥8 (range from 8 to 12, mean 10) 3q signals (range 54%–66%, mean 49% neoplastic nuclei) (Fig. 2). Twenty out of 40 (50%) showed from 3 to 7 signals (mean 6) (Fig. 3). The remaining showed two fluorescent signals per nuclei (30%). When corrected by whole chromosome 3 signals, only cases with ≥8 signals maintained a LSI 3q/CEP3 ratio >2. Overall, FISH findings are summarized in Fig. 4.

### Array CGH findings

Among 6 cases tested and focusing on the 3q region, DNA copy number changes were found in two cases (+3q25.3-q27.3). The whole aberrations span about 27.5 Mb. Specifically, findings from oligonucleotide probes spanning the SOX2 and PI3CA zones revealed more relevant gains for the PA3CA in respect to the SOX2 zones, although significant differences were not observed.

The remained cases had normal profile. Relevant genomic abnormalities were observed in three cases (+5p).

When matching cases analyzied by both FISH and aCGH, only those two cases showing 3q amplification by aCGH scored ≥8 fluorescent at FISH and evidenced a 3q/CEP3 ratio >2. The remaining four cases showed a flat genomic profile at aCGH with focus on the 3q and centromeric regions.

## Discussion

In our study we observed that 1) absolute copy number of 3q chromosomal region may harbour false positive interpretation of 3q amplification in squamous cell carcinoma; 3) correction for chromosome 3 is important to identify true 3q amplification; 4) we propose to consider a case “amplified for chromosome 3q” when scoring ≥8 fluorescent signals; 5) *false* versus *true* 3q chromosomal amplification in squamous cell lung carcinoma merit consideration at light of emerging trials involving anti-PI3CA and SOX-2 targeted drugs.

Amplification of chromosome 3q has been described in squamous neoplastic transformation from different sites [25]. Althought abnormalities on chromosome 3 and 3q has been observed in minority percentages of lung adenocarcinomas with different rates, in the squamous cell lung carcinomas amplifications is frequently detected at 3q24–q27 and represent a sensitive genotypical marker of the squamous differentiation of lung carcinomas [5], [26], [27], [28], [29], [30], [31].

In our study we highlighted the findings of three distinctive clustered cytogenetic patterns and propose that correction for chromosome 3 is critical for the determination of true 3q amplification status. Today, there is no definitive consensus about the optimal scoring system for assessing 3q amplification. Due to the fact that the 3q region maps genes, such as SOX2 and PI3CA genes, that may have potential role as novel targets for therapeutics, we believe that the need in proposing guidelines for appropriate evaluation of the 3q region status is important and may have impact to different clinical levels. The absolute 3q copy number or the alternative 3q/CEP3 ratio has to be evaluated appropriately, when the predictivenesss to drug efficacy is going to be determined. In our serie eight cases displayed true amplification (≥8 fluorescent signals per nuclei) when corrected by chromosome 3. We validated these findings after wide genomic aCGH analysis. False positive 3q amplification is present when absolute copy number of 3q signals ranges from 3 to 7 per nuclei. Wide genomic molecular profiling such as the array comparative genomic hybridization technique may help in appropriate definition of new cut-offs and validate standard ISH results and interpretation. aCGH may distinguish gains of a chromosome due to polyploidy or genetic instability versus true chromosomal amplification.

The chromosomal region spanning the 3q encounters many interesting genes [32], [33]. Several potential targets in the 3q region, particularly at the 3q26–28 amplicon, have been identified [6], [34], [35]. More recently, the knowledge of the key role of SOX2 oncogene which maps specifically to the distal part of chromosome 3q increases importance [19], [20], [21]. Wilbertz et al. found that SOX2 amplification and upregulation are frequent events in squamous cell carcinomas of the lung and are associated with indicators of favorable prognosis [36]. Hussenet et al. highlighted the recurrent SOX2 activation and its necessary role for squamous neoplastic cell survival in squamous cell carcinoma [37].

At routinely laboratory level, fluorescence in situ hybridization (FISH) on formalin-fixed, paraffin-embedded tissue is an increasingly useful technique in the detection of many diagnostic chromosomal abnormalities in different oncological fields [38].

Tonon et al. observed and highlighted different degree of gains of chromosomes/genes in term of magnitude such as focal copy number alterations, such as five genes gained in lung carcinomas and others recurrent copy number alterations including high-amplitude amplifications [39]. McCaughan et al. observed in a subset of squamous lung carcinomas that progression of high-grade preinvasive disease is associated with incremental amplification of SOX2 and concluded that progressive 3q amplification is present in the evolution of preinvasive squamous cell carcinoma. Authors proposed the SOX2 gene as a key drug target of this dynamic process [8]. Pelosi et al. focused their study on preneoplastic/preinvasive squamous cell proliferations and early-stage invasive carcinomas of the lung. 3q26 amplification and polysomy of chromosome 3 were confined to malignant samples, with 37% of invasive squamous cell carcinomas, and 27% of severe dysplasias/in situ carcinomas showing these chromosomal abnormalities [40].

The PI3K gene also set in the 3q chromosomal region. There are ample genetic and laboratory studies that suggest the PI3K–Akt pathway is vital to the growth and survival of cancer cells [41]. Inhibitors targeting this pathway are entering the clinic at a rapid pace and the therapeutic potential of drugs targeting PI3K–Akt signalling for the treatment of cancer is under deep consideration [41]. Targeting PI3K gene in squamous cell carcinoma with 3q amplification may be rationale-based promising trials.

In our study we did not observe relevant differences in term of magnitude of genome gains when analyzing findings from oligonucleotide probes spanning the SOX2 and PI3CA chromosomal regions.

The clinical impact of the multifaceted genotypic abnormalities, observed in the actual study, merits further investigation. We evidenced that absolute enumeration of chromosomal copies is challenging and the absence of analytical standardisation may limit pre-clinical and clinical studies focusing on the predictiveness of biomarkers mapping on the 3q region. False *versus* true 3q chromosomal amplification in squamous cell lung carcinoma are observed and its impact to trials involving anti-PI3K and -SOX2 targeted drugs is a direct consequence. Correction for chromosome 3 is critical for the determination of true 3q amplification status: the definition of a detailed scoring system and a consensus is mandatory.
